# Dental Technic

**Published:** 1898-09

**Authors:** J. Quincy Byram

**Affiliations:** Indianapolis.


ARTICLE VIII.

### DENTAL TECHNIC.

DR. J. QUINCY BYRAM, INDIANAPOLIS.

**Read before the Indiana State Dental Association.**

Methods of teaching are always changing, and those taught to-day are soon to be discarded for more improved and scientific ones. Technic teaching being one of the latest methods of teaching first year students in dental colleges, offers many interesting points to the profession. The course as outlined in the paper may not be the best, but is one that proved very satisfactory in the Indiana Dental College.

The majority of students that enter a dental college are from the farm, the shops, the mercantile counters or the school, and know nothing of dentistry. Many of them do not know a chisel from an excavator or a vulcanite scraper from a scaler. On the other hand, there are a few who have been in offices long enough to gain some valuable experience.

To-day when a pupil enters a public school he is taught by the objective method. The best teachers recognize that a child gains a much better understanding by seeing and handling and doing than by merely hearing and remembering. So his work has to do with concrete objects which represent the ideas that he seeks to master. By associating these objects with the idea, his memory develops better and faster. The tendency is to carry this work farther, and as he advances, a course in manual training is offered. Manual training is not only education of the hands, but also by the hands; and a systematic training of the hands is not antagonistic to mental growth, but assists it.

The surgeon must have a practical knowledge of the human organism; he gains this by studying the practical along with the theoretical. The pathologist studies the diseased tissue along with the healthy. We would not think of studing chemistry without making practical tests of the compounds studied. By instruction alone the artist would never be able to blend his colors on the canvas, but must be constantly using his brush. The same is true in all the branches of science and art.

The development of the dental student should be three-fold: (1) The development of the intellect or the theoretical; (2) the development of the touch or the practical; (3) the development of the ethical or sense of duty. The relation of each to the other is such that they are inseparable.

Until recently the great question among colleges has been what to do with first-year men. With the increasing demand for a more scientific knowledge there grew a tendency to extend didactic teaching at the expense of the practical. The students receive lectures on operative and prosthetic dentistry. Taking it for granted that they remember all they hear, it will be at least two months before they have gained enough knowledge for it to be of practical value. They have no knowledge of dental anatomy; can hardly tell a root from a crown, and do not know a cavity from a fissure. They cannot be crammed full in a few days. Their fingers cannot be well trained on living tissues. It is almost impossible to demonstrate on patients so that the students can understand, for no two will be doing the same operation at the same time. No college can keep a staff of demonstrators large enough to satisfactorily teach them in a systematic way. The greatest disadvantage is the impossibility of keeping them busy. It is impossible to keep from fifty to eighty freshmen at work on patients all the time. The old adage that the best way to keep a boy out of mischief is to keep him busy, applies to the dental student. To say the least of the old system, it was unjust to the student and inhuman to the patient.

Dental technic fills this gap and give a systematic course of study for the first-year students. Of this system of teaching, Dr. E. C. Kirk says: "The principle involved in the technic system of instruction is broader and means more than mere cultivation of the brain and hand. It includes the use of all the perceptive faculties as means of brain cultivation. By the application of this principle not only may manual dexterity be achieved, but all the powers of observation be trained to their highest capacity, and the reasoning faculty correspondingly developed."

This course is taught independent of the operative and prosthetic dentistry, although some of the work which has been given under these chairs may be reviewed. While an effort is made to develop along all the lines mentioned, special effort is given to develop the sense of touch; also, to familiarize them with their work, so that when they begin operations for patients, they can see the finished work with the mind's eye.

First a lecture on plaster of paris is given. They must experiment and learn what effect the various salts and hot water have on it. After a lecture on impressions, they are paired off to take impressions of each other's mouths. Next the models are constructed; after separating, a wax base plate is made for the palate, the teeth are properly trimmed, the case flasked, packed, vulcanized, finished and fitted to the mouth, each step having been taught them before they proceed. This gives them an idea of the process of constructing a denture, and also accustoms them to the manipulation of plaster of paris.

After a series of lectures on steel and making instruments, they make several instruments which not only have the proper shape and size, but must stand the tests for spring and temper. Some, after ruining several blanks, learn that they are only blacksmiths on the small scale, and not very good ones at that. These instruments will be useful to them all through the course.

A varied course is more interesting to them, and keeps the work from becoming irksome. Occasional talks are given on dental anatomy, which is divided into descriptive and structural. Under descriptive especial attention is given to nomenclature and terminology; form and arrangement under structural to microscopic.

The simplest system of nomenclature should be taught. Only one term to express the same object should be used. Such words as canine, stomach and eye-tooth are dropped, and only cuspid used. Also the terms sixth-year, twelfth-year molars and wisdom tooth are dispensed with, and the terms first, second and third molars used instead.

Another important change is the simplification of terms expressing the surfaces and location of teeth. Let mesial, distal, labial, lingual and occlusal designate the surfaces of all teeth. By so doing we do away with such terms as anterior, posterior, front, back, palatal, grinding, cutting, etc.

Microscopic anatomy is taught by lectures and dissection of teeth. The student is required to draw and describe the tooth, locate such point of interest as tubercles, cusps, fossæ, grooves, fissures, and sulci. As soon as they are familiar with the surfaces of the crown and roots, they make a series of cross and longitudinal drawings designating the shape and size of pulp chambers and root canals. For convenience the root is divided into five sections: (1) Apical, (2) apical ⅓, (3) middle, (4) gingival ⅓, (5) gingival.

After they become familiar with the pulp chamber and root canals, one of each group of teeth is invested in plaster for root canal filling. First they are taught both radical and conservative pulp treatment; also, how and when to devitalize pulps. Different systems of root canal filling are taught. Some are cleansed and filled immediately, while others are treated with various antiseptics before filling. A number of materials are used for filling the canals. After all the canals are filled, the teeth are dissected and the students designate by drawings just how each one was filled. By this time their touch is lightened and their knowledge of a tooth is sufficient to enable them to understand the principles of filling. To develop these principles by the objective method, the vulcanite tooth form suggested by Dr. Weeks is used. Only hand instruments are used for excavating. Cavities of all classes are excavated on the surfaces of the base of the form and are filled with tin and plastics. The tin is used both as cohesive and non-cohesive.

Now that their touch is becoming lighter, they begin work on the teeth. The cavities are divided into three classes. The first class: Those caused by structural imperfections, and coming in contact with secretions of diseased tissue. They are shown the necessity of following fissures to the end and making fillings self-cleansing. The second class: Those cavities on the approximal surfaces of incisors and cuspids. This class is subdivided and each division studied separately. There they are taugh the necessity of preserving the interproximate space. The third class: Those cavities on the approximal surfaces of bicuspids and molars. This class is also subdivided. Large models of teeth made of clay are used to demonstrate with, and cavities of each class are prepared and filled before the students begin preparing their cavities.

At convenient times about ten at a time are taken to the infirmary and shown how to adjust the rubber dam. They then adjust the dam to each other's mouths, with and without ligatures; also with rubber dam clamps.

For crown work they take partial impressions of each other's mouths, construct their models, and make plaster articulators. Only band crowns are made, and each student must carve the teeth used for making impressions for running dies. In making bicuspids and molars the band is fitted to the tooth on the model. The cusps are made by filling the band with modeling compound and closing the antagonizing teeth. The band and modeling compound is removed and the cusps are carved to required shape. Great care is taken not to disturb the depressions made by the antagonizing cusps. This is used to make an impression in clay for making dies. This system is used because it requires the students to be accurate and keeps a good articulation before them all the time.

A number of cast models are articulated to use in taking impressions for full denture. The inferior is taken in plaster and the superior in modeling compound. After the models are constructed they articulate the case by the base-plate method. The teeth are articulated and the case carried through all the steps to completion. As soon as the full denture is completed they again take impressions of each other's mouths. When the models are obtained, two or more teeth are trimmed off. They make a partial replacing of the teeth.

So far I have dealt only with the theoretical and practical teaching. It is not the work of a technic teacher to give a course of lectures on ethics; but it is his duty to make every lecture and every demonstration such that the student will feel that he has chosen a worthy profession—a profession where he must use his concentrated energy to be successful; that there is more for him to do than merely to attend three courses of lectures in some college. He must be taught to be conscientious and to do his very best on every operation, that he will receive reward for good work and disapproval for inferior. If a student is required to work hard three hours each day for six months, he begins to see that it takes work to succeed. There are but few who do not have enough pride to impel them to move forward and try to keep them from being the drones of the class.—*Indiana Dental Journal.*

